# Psychosocial implications of rare genetic skin diseases affecting appearance on daily life experiences, emotional state, self-perception and quality of life in adults: a systematic review

**DOI:** 10.1186/s13023-023-02629-1

**Published:** 2023-02-23

**Authors:** Hugo Fournier, Nicolas Calcagni, Fanny Morice-Picard, Bruno Quintard

**Affiliations:** 1 Laboratoire de Psychologie (LabPsy) EA4139, Univ. Bordeaux, 3 ter Place de la Victoire, Bâtiment A - 1er étage, 33000 Bordeaux, France; 2 CHU Bordeaux, 33404 Bordeaux, France

## Abstract

**Background:**

Since the beginning of human genetic research, there are very few publications sharing insights of the negative impact of rare genetic skin diseases (RGSD) on patients’ experiences. This systematic review assessed the psychosocial implications of these conditions in terms of daily life experiences, emotional state, self-perception, and Quality of Life (QoL).

**Methodology:**

A systematic review was carried out on albinism, neurofibromatosis type 1 (NF1), birthmarks and inherited ichthyosis. The PubMed, Scopus, PsycArticle, PsychInfo, Psychology and Behavioral Sciences Collection, and SOCindex databases were queried. Inclusion criteria were adult patients with one of these RGSDs. Simple descriptive statistics and qualitative content analysis were conducted to summarize the main results reported by the authors.

**Results:**

Of the 9987 articles retrieved, 48 articles were included: albinism (16), NF1 (16), inherited ichthyosis (10), birthmarks (6). The majority of the studies on albinism were conducted in Africa. Twenty-seven studies quantitatively assessed diverse psychological parameters: 13 showed a significant impact of the disease on QoL, five on emotional state, two on self-representation and two others on psychiatric comorbidities. Disease severity and visibility were good predictors of QoL (except for albinism). Body image and appearance concerns were also associated with QoL and emotional state. The 19 qualitative studies highlighted recurring themes across each of these diseases: discrimination and stigma during childhood and adolescence, discomfort in social interactions, guilt of transmission, the importance of social support from family and friends, altered daily life functioning, altered romantic and sex life, limited academic and professional aspirations, lack of interest and support from the medical field, and the unpredictability of the evolution of the disease. The only two mixed-method studies in this review were unable to contribute to any inferential analyses but could corroborate some of the qualitative findings.

**Conclusion:**

These results showed that RGSDs have a significant impact on different aspects of patients’ lives. This review has demonstrated that there is a real need for support systems for patients with these diseases. Such systems should be developed to provide them with necessary information and to guide them through an appropriate care pathway.

**Supplementary Information:**

The online version contains supplementary material available at 10.1186/s13023-023-02629-1.

## Introduction

Formerly known as “orphan diseases”, rare diseases include a wide range of conditions: physical, sensory, mental, visible, or invisible; are often progressive, sometimes leading to permanent disability; and are most often without cure. In Europe, they are characterized by a prevalence of less than 1/2000 and are mostly of genetic origin (about 80%). There are between 6000 and 8000 rare genetic diseases in the world and, when combined, they affect 6–8% of the population. Extrapolating these data to the European population, between 25 and 30 million people could be affected on the continent [1–3]. In this respect, rare diseases, when grouped together, are no longer truly so and constitute a real health issue that is still not sufficiently studied in the psychosocial field.

As mentioned above, some of these diseases are visible and are responsible for their own set of difficulties. This is especially true for rare genetic skin diseases (RGSDs). Among them, we will focus more specifically on albinism, neurofibromatosis type 1 (NF1), inherited ichthyosis (vulgaris, lamellar, harlequin etc.), and the particular case of congenital birthmarks.^1^

“Albinism refers to a group of rare congenital diseases globally characterized by poor vision and a variable hypopigmentation phenotype” [4]. Albinism and its many forms have been widely investigated in genetics^2^ [5–11], but psychosocial research on albinism life experiences is very limited and the results are often not generalizable [12]. In addition, many of the publications on albinism are in the form of essays, testimonies, or appeals [13–18]. On the African continent, people with albinism (PWA) are the target of violent discrimination associated with multiple beliefs and superstitions [19–22]. Nevertheless, several studies have also looked at young PWA’s life experiences in terms of education [23–27], social functioning [28, 29], and self-concept [30]. Moreover, the last decade has seen a significant amount of research emerge in health psychology regarding adults with albinism. [31–45]

“Neurofibromatosis type 1 (NF1) is a clinically heterogeneous, neurocutaneous genetic disorder characterized by café-au-lait spots, iris Lisch nodules, axillary and inguinal freckling, and multiple neurofibromas” [46]. NF1 has already been the subject of numerous studies in psychology, particularly in a child or adolescent's development [47–58] but also in their parents' experiences [59–67]. Moreover, numerous studies have sought to establish a link between neurofibromatosis and atypical developmental profiles such as autism spectrum disorders [68–77] and attention deficit disorders [68, 78–80] in order to explain learning difficulties and social functioning. Other studies have also shown that NF1 has a significant impact on a young person's quality of life (QoL) and on their family functioning [72, 81–86].

Inherited ichthyoses, also known as disorders of keratinisation (DoK), encompass a heterogeneous group of skin diseases that are characterized by extremely dry and rough skin and the presence of an excessive amount of dead skin flakes (squames) that are continuously shed. The severity is variable. The skin is thick and inflammed, sometimes with painful cracks or blisters [87, 88]. Again, over the past 10 years, a number of studies have emerged on the impact of ichthyosis on the QoL of affected adults and children [89–97].

Concerning the particular case of congenital birthmarks, and more specifically here, port-wine stains (PWS or angiomas), these are vascular malformations that affect the capillaries of the skin. They appear as purplish red spots which have an aesthetic impact when they are located on visible areas (face, neck etc.) [98]. They are present from birth and persist throughout life, however they can be treated with pulsed dye lasers to achieve significant lightening [98–100]. Despite the limited amount of research dealing with the experiences of people with PWS, some recent studies have shown a renewed interest in this topic by focusing on the QoL of these patients [101, 102]. The main problem, however, is a significant methodological heterogeneity among these studies.

Despite this growth of research into the psychosocial consequences of rare genetic diseases in the last two decades, data remains very sparse and does not provide a good understanding of how these conditions may be experienced by adult patients. In this context, our objective for this systematic scientific literature review is to investigate the psychosocial implications of an RGSD on daily life, emotional state, self-perception and QoL in adults. Three main questions will guide our research strategy: 1) What is the impact of RGSD on daily life, emotional state (anxiety, depression, well-being), self-perception, and QoL? 2) What are the main predictors of QoL, emotional state (anxiety, depression, well-being) and self-perception of patients with an RGSD? 3) What psychosocial aspects do these RGSDs have in common (i.e., predictors, consequences, functioning)?

## Methods and materials

PRISMA [103] and INESSS [104] guidelines were followed throughout the present review.

### Eligibility criteria (PICOTS)

#### Population

Studies including adult (> 18 years) patients with a diagnosis of a genetic skin condition affecting appearance, specifically birthmarks (PWS, congenital melanocytic nevus), albinism (oculocutaneous albinism, Hermansky-Pudlak syndrome), inherited ichthyosis (vulgaris, lamellar, harlequin etc.), and NF1.

#### Intervention

We only focused on observational studies. All intervention-based studies identified have been excluded. For reasons of overall consistency, we did not include psychosocial studies conducted after a specific treatment (e.g., pulsed dye laser for PWS), which seek to determine the effect of the intervention on patients’ overall outcomes.

#### Comparison groups

All studies, with or without one or more comparator groups, were included. Thus, all studies that compared RGSD subjects to the general population, chronic health populations or other populations with appearance-altering conditions were considered in this review.

#### Outcomes

The type of results considered in this review were:

1. Data that accounted for the impact of genetic skin conditions affecting appearance on QoL, emotional state, or self-perception.
2. Psychosocial factors that may be associated with QoL, emotional state, or self-perception in patients with genetic skin conditions affecting appearance.
3. Aspects of the participant's daily life that are particularly affected by their genetic skin condition.

#### Temporality

This criterion is not binding. Cross-sectional and longitudinal design were both considered with the same interest.

#### Settings

Studies using qualitative methods (based on semi-structured interviews or focus groups) and quantitative methods (based on numeric scores via questionnaires and scales) have been considered. They could be set as cross-sectional data collection carried out within the healthcare structure (face-to-face interviews, questionnaires completed on site) or remotely (phone interviews, online surveys).

This review has been restricted to English and French language, peer-reviewed, and indexed studies. Conference abstracts, literature reviews, academic dissertations, and case studies have been excluded. We didn’t set any inclusion date because, considering the other criteria we established, we didn’t want to take any risk of omitting interesting results by limiting ourselves to arbitrarily defined publication dates.

### Information sources

We queried the following electronic databases: Medline (via PubMed), Scopus, PsycArticle, Psychinfo, Psychology and Behavioral Sciences Collection, and SOCindex (via EBSCO). The search was started in February 2020 and the last update was in May 2022.

### Search algorithm

Keywords included successively in all databases were:

**Table:** 

"skin genetic disorders" OR "port-wine-stains" OR "giant congenital melanocytic nevus" OR albinism OR "Hermansky-Pudlak" OR "Chédiak-Higashi" OR "neurofibromatosis type I" OR "Von Recklinghausen" OR Ichthyosis OR "ichthyosis vulgaris" OR "lamellar ichthyosis" OR "ichthyosis bullosa of siemens" OR "X-linked ichthyosis"	1
AND	
Affect* OR Adjustment OR Adaptation OR Acceptance OR Satisfaction OR Happ* OR Optimis* OR Well-being OR Depress* OR Anxi* OR Mood OR Distress OR (Social AND Fuctioning) OR "Quality of life" OR QoL OR Appearance OR Disfigurement OR (Patient AND Satisfaction) OR Factors OR Expect* OR Impact OR (Disease AND Related AND Disability) OR (Quality AND Care) OR Psycho* OR Emotion* OR (Psychological AND Distress) OR (Psychological AND Stress) OR (Psychosocial AND Need) OR (Common AND Mental AND Disorder) OR (Stress AND Disorder*)	

The search strategy outlined above was adapted if (a) too few results were returned, suggesting that some relevant literature may have been missed; or (b) if too many results were found, rendering a meaningful search unfeasible. The first author performed all database searches.

### Study selection

**Fig. 1:**
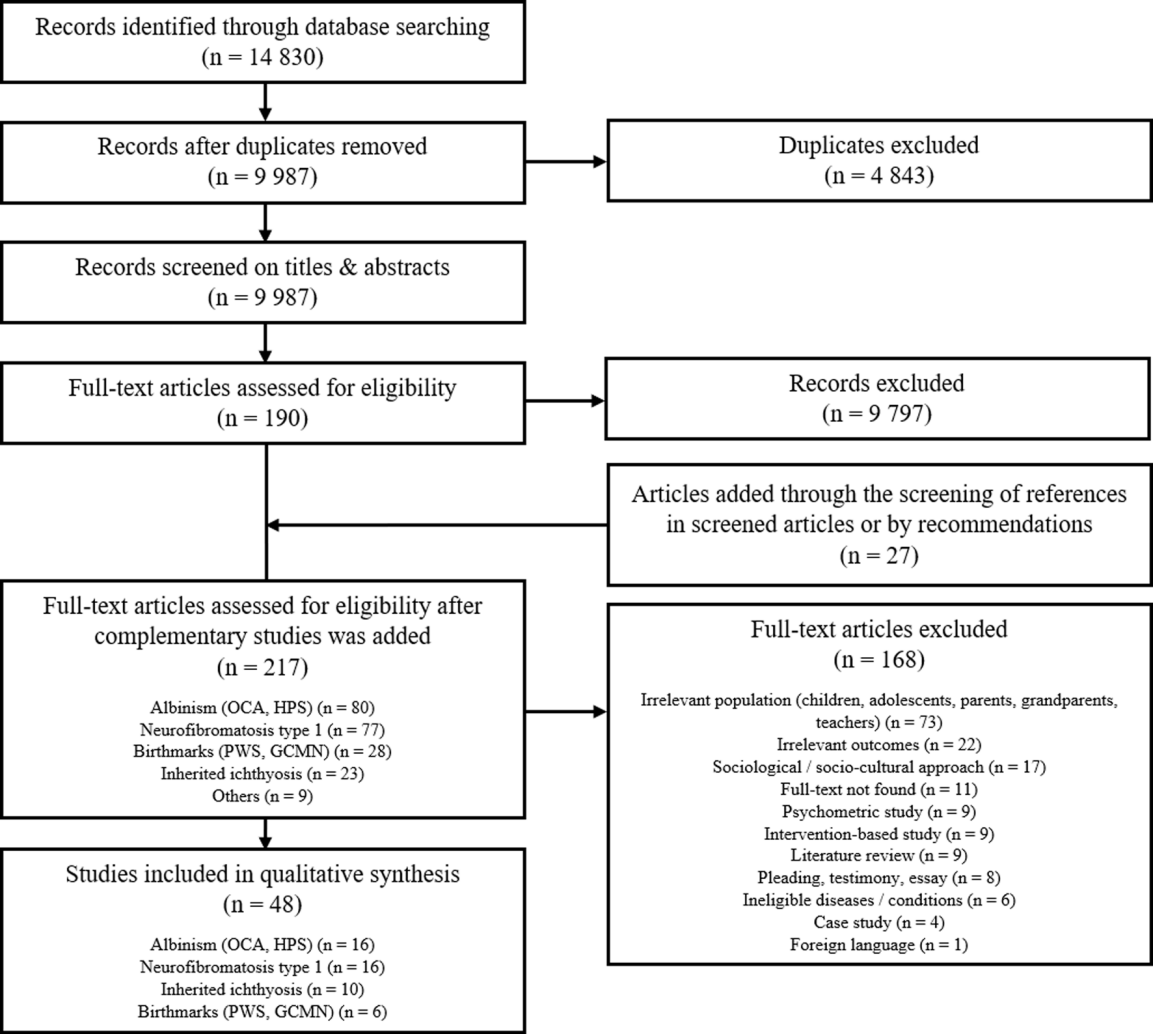
Flow diagram of included studies

Retrieved titles and abstracts (saved on Zotero) were systematically screened to determine possible study eligibility. Any summaries referring to one of the diseases of interest associated with QoL; psychosocial aspects, implications, or consequences; emotional state (anxiety, depression, well-being); and appearance, body image, or self-conception (self-esteem) were included in the full review of the article. Studies were included in the final review when eligibility criteria were confirmed after reading the whole article: adequate population, design, and relevant outcomes. They were excluded when either an irrelevant population (e.g., aged below 18, being family members of PWA, etc.), a study design that relied on a sociological or socio-cultural approach, or irrelevant outcomes (bio-medical parameters, cognitive or intellectual functioning etc.) were present. [see Flowchart—Fig. 1].

### Data items and collection process

The extraction of information from the selected studies was based on the recommendations of INESSS (2013). For each article, information about design, participant characteristics (sample size, age range, gender, kind of disease, etc.), locations (geographical areas), methods used (control strategy, measurement tool, data analyses), significant outcomes, and limitations had to be systematically noted. These data were manually identified in each study and noted on a spreadsheet. The first author (HF) randomized one third of the articles included for the full-text step and gave those to the second author (NC) in order to realize a dual independent selection, inclusion, and data extraction. In addition to the data mentioned above, each of the two researchers had to state their decision regarding the inclusion of each article, stating their arguments for and against. All articles in which the researchers had doubts about their inclusion were placed in a category "to be discussed collectively". Then, HF and NC pooled their results, and discussed with the other authors in order to validate the studies that had been individually included and to reach a consensus on the uncertain articles.

### Summary strategy

Simple descriptive statistics and qualitative content analysis were used. As our studies showed clinical heterogeneity in terms of settings (sample size, kind of diseases, locations), study design, methods (measurement tools, control strategy) and outcomes, it was impractical to run a quantitative analysis for a meta-review. However, we have synthesized the main data from each study in tables constructed from those proposed by the Public Health Agency of Canada (2014) [105], the INESS guidelines (2013) and Calcagni et al. (2019) [106]. An analytical narrative synthesis was conducted to develop an in-depth understanding of the common and specific psychosocial implications of each identified disease (INESSS 2013).

### Risk of bias

The Control Guidelines Critical Appraisal Tool Kit (developed by the Public Health Agency of Canada, 2014) [105] was used to independently rate the quality of each quantitative study and to allocate a specific grade (see Appendix). Concerning the qualitative studies, we built our own evaluation grid based on the one developed by the Public Health Agency of Canada (2014) and the work of Santiago-Delefosse (2004) [107] and Stenfors, Kajamaa & Bennett (2020) [108] [see Additional file 1: “Quali study assessment grid”]. Thus, grid selection was adapted, and assessment criteria weighted according to the design of each study. For each design, as some of the scoring criteria are based, in part, on the subjectivity of the first author, the percentages awarded have been transformed into an appreciation corresponding to the interval within which the score is included: High (100–75%), Moderate (74–40%), Low (40–0%). The studies were assessed on the basis of two indicators: one exclusively based on the strength of the study design, the other on the overall article quality (i.e., sampling, internal validity, ethics, etc.). To maximize study inclusions, we did not use a minimum cut-off score as an inclusion criterion.

## Results

### Study selection

A total of 14,830 references were found, including 7019 through PubMed, 6971 through Scopus, 731 through PsycINFO, 59 through Psychology and Behavioral Sciences Collection, 35 through Socindex, and 15 through PsychArticle databases. All citations were exported to the bibliographic management software Zotero to facilitate the processing of all records. After excluding duplicates (*n* = 4843), 9987 references were screened (titles and abstracts), which led to the exclusion of 9797 studies. Furthermore, 27 articles were added through the screening of the reviewed studies or by recommendations. Thus, 217 full-text articles were assessed, of which 168 were excluded, leaving 48 studies. (see Fig. 1 for explanations).

Before going any further, it is important to point out that we recorded three recently published literature reviews specifically dedicated to inherited ichthyosis [89] and PWS [109, 110]; they included studies that focused on children and adolescents and also examined psychosocial intervention-based studies. As we cannot include these articles in our current review, we will consider their results in the discussion and see if their findings are consistent with ours.

### Overall considerations (all diseases)

A total of 3703 participants were included across all studies. As the present studies are based on two very different methodological frameworks, we will separate the results of qualitative exploratory studies from quantitative cross-sectional studies in the following section (see Table 1).

**Table 1:** Synthesis of data from reviewed articles, all diseases combined, according to their number, study design, samples’ main characteristics (number of subjects, average size, location), comparator strategies and main outcomes

	N	Design (n)	Sample				Comparator.s (n)	Main outcomes (n)
			Total	M (sd)	Range	Locations (n)		
Overall	48	Quanti (27)	3265	121,89 (66,67)	26–244	Europe (12) North America (7) West Africa (4) South America (1) East Asia (1) Australia (1)	Database.s extraction.s (15): Non-affected (8) Other disease.s (3) Both (4) Recruited sample (5): Non-affected (3) Other disease.s (2) N/A (9)	Impaired Quality of Life (global, health related, skin specific) (14) Worse emotional state (anxiety, depression, well-being) (7) Disease severity impact (6) Disease visibility impact (5) Appearance self-awareness / Body image or related attitudes (4) Self-esteem (2) Psychiatric comorbidities (2)
		Mixed (2)	23	11,5	10–23	Europe (1) South America (1)	Non-affected (1) N/A (1)	Vulnerable skin Discrimination / stigma during childhood and adolescence (teasing, bullying) Discomfort in social interactions (dealing with strangers’ reactions) Transmission guilt-trip / genetic inheritance concerns Importance of social support (family, friends, patient’s community) Altered daily life functioning (mobility, skin self-care) Altered romantic & sex life Limits academic & professional aspirations Lack of interest and support from the medical field Unpredictability of the disease evolution Parental distress / investment
		Quali (19)	415	21,32 (16,15)	3–62	Europe (5) Southern Africa (4) North America (2) Caribbean (2) West Africa (2) Central Africa (1) Middle East (1) East Asia (1) Australia (1)	Recruited sample (1): Non-affected (1) N/A (18)	
Albinism	16	Quanti (6)	430	75,83 (27,05)	38–105	West Africa (4) Southern Africa (1) South America (1)	Recruited sample (4): Other disease.s (2) Non-affected (2) N/A (2)	Worse emotional state (anxiety, depression, well-being) (3) Psychiatric comorbidity (1) Impaired Quality of Life (1) Social support (1) Stigma (1)
		Quali (10)	199	19,9 (17,18)	3–62	Southern Africa (4) Caribbean (2) West Africa (2) Central Africa (1) East Asia (1)	Recruited sample (1): Non-affected (1) N/A (9)	Others’ attitudes / discrimination, stigma (7) Health care gaps (4) Importance of social support (family, community) (4) Superstitions & beliefs (4) Knowledge about albinism (3)
Neurofibromatosis 1	16	Quanti (8)	1009	126,13 (61,38)	37–228	Europe (5) North America (3)	Database.s extraction.s (6): Non-affected (4) Other disease.s (0) Both (2) Recruited sample (3): Non-affected (2) Other disease.s (1) N/A (1)	Impaired Quality of Life (health related, skin specific) (5) Disease visibility (4) Self-esteem (2) Worse emotional state (2) Appearance self-consciousness/Body-image (2) Psychological distress (1) Loneliness (1) Disease severity (1)
		Mixed (1)	13	–	–	South America (1)	Non-affected (1)	Affected body-image / self-consciousness (6) Affected aspiration, self-esteem & self-confidence (5) Social functioning (5) Unpredictable disease progression (5) Pain (4) Knowledge about NF1 (4)
		Quali (7)	158	22,57 (18,21)	6–60	Europe (3) North America (2) Middle East (1) Australia (1)	N/A (7)	
Ichthyosis	10	Quanti (8)	1193 (including about 270 subjects < 18 yrs)	149,13 (71,73)	26–241	Europe (5) North America (3)	Database.s extraction.s (2): Non-affected (1) Other disease.s (0) Both (1) Recruited sample (1): Non-affected (1) N/A (5)	Impaired Quality of Life (5) Disease severity impact (4) Worse emotional state (2) Family burden (1)
		Mixed (1)	10	–	–	Europe (1)	N/A (1)	Body discomfort (2) Time-consuming skin care (2)
		Quali (1)	25	–	–	Europe (1)	N/A (1)	
Birthmarks	6	Quanti (5)	634 (including about 10 adolescents)	126,8 (87,46)	52–244	Europe (2) North America (1) East Asia (1) Australia (1)	Database.s extraction.s (3) Non-affected (1) Other disease.s (1) Both (1) Recruited sample (1): Other disease.s (1) N/A (1)	Impaired Quality of Life (3) Body image / attitudes (2) Psychiatric & medical comorbidities (1) Social support (1) Disease severity impact (1) Disease visibility impact (1) Worse emotional state (well-being) (1)
		Quali (1)	23	–	–	Europe (1)	N/A (1)	

A total of 3265 subjects were included in quantitative protocol (mean = 121.89); by contrast, 415 were included in exploratory qualitative research (mean = 21.32). Only two studies were built on a mixed design and included a total of 23 subjects. Over half of the included studies were led in high income countries (European countries, *n* = 18; USA, *n* = 9; Australia, *n* = 2), except those concerning albinism, of which 3/4 were led in African countries (*n* = 11). Of the 48 studies selected, 11 also included participants under the age of 18 in their samples (22.92%). For this review, we were only interested in the results concerning adults (which are often compared with the results observed in children and adolescents). It can also be noted that quantitative studies have a greater tendency to mix age groups within their samples than in the qualitative studies we have selected (quan., *n* = 9 vs. qual., *n* = 2), and especially in research on ichthyosis (*n* = 5). In addition, two qualitative studies also included relatives in addition to affected participants. Of the 27 quantitative studies included, 20 used comparators as well as the mixed study, either by recruiting unaffected participants, by creating groups of patients with another disease, or by extracting data from previous research.

Concerning quantitative results, a significant impact of rare skin genetic disorders on QoL was observed in 14 of the 18 studies measuring this variable (global, health-related, and skin-specific form). NF1 and ichthyosis are the most documented diseases in this respect (for each, *n* = 5), followed by birthmarks (or PWS, *n* = 3), and finally albinism (*n* = 1). Furthermore, QoL was often associated with the level of severity and/or visibility of the skin disorder (*n* = 9, albinism studies excluded). In the same vein, some studies that focused on NF1 and birthmarks have linked body image or appearance self-consciousness with QoL, psychological distress, emotional state, or even self-esteem (*n* = 4).

On the qualitative side, a wide range of results could be observed. Most of these studies are based on more or less structured individual interviews (*n* = 16, + 2 including the mixed studies), one was based on focus groups, another on mixed individual interviews and focus groups, and the last one on writing an essay about the participants' condition.

Regarding the quality of the studies, the first observation that can be made concerns the great heterogeneity of the methods and study designs. Among the 19 qualitative studies, 11 were judged to be of high quality, four of moderate quality, and four of low quality. Of the 27 quantitative studies, eight were of high quality, 18 of moderate quality, and only one of low quality. The strength of the designs was globally weak because we did not include any experimental or intervention-based studies (considered as designs that can provide a higher level of evidence). As qualitative studies cannot generalize their findings, all qualitative designs were judged to be low.

Regarding outcomes, even if there are common themes between the four conditions of interest, there are significant specificities, depending on the disease itself, but also on the context in which the subjects live. In the following sections, we will explain these results, disorder by disorder, and finally, propose a synthesis on the lived experiences of patients with RGSDs (common core to specific aspects).

### *Research on albinism: n* = *16*

A total of 654 participants were included across albinism-related studies. As mentioned earlier, most of the studies were carried out on the African continent (Nigeria, *n* = 5; Botswana, *n* = 2; South Africa, *n* = 2; Malawi, *n* = 1; Cameroon, *n* = 1; Ghana, *n* = 1) because this genetic condition is more widespread in Africa than in Europe or North America (UE & NA = 1/20,000 vs. AF = 1/5000–15,000) and the psychosocial repercussions it entails are much more serious, as we will see later in this section. The other research were conducted in Taiwan (*n* = 1), Brazil (*n* = 1) and Puerto Rico (*n* = 2). Sixty nine percent of the studies included mostly female participants (6% female-only participants, 63% more than half female participants). The sex ratio is balanced in four qualitative studies [31–33, 111], with men outnumbering women in only one study [34]. Overall, women represent 57.65% of the subjects in all of the samples. The average age could not be calculated because this information was missing in two articles [34, 35]. Two also included participants under 18 years old in their sample [34, 36].

On the quantitative side, six studies were conducted involving 430 subjects (mean = 75.83). On the qualitative side, 10 studies have been included involving 199 subjects (mean = 19.9). Globally, they showed that PWA see albinism as a source of problems and a disadvantage compared to non-affected people (Table 2).

**Table 2:** Details of reviewed studies about albinism, according to location, sample, method design and significant outcomes

Setting			Method					Results	Study quality
Ref.	Location	N	Design (strength)	Tools	Comparator(s)	IV(s)	DV(s)	Significant outcomes	
Anshelevich et al. [37]	Botswana	27	Exploratory -qualitative (low)	Semi-structured interviews (TCA)	N/A	N/A	N/A	Educational environments: health-related barriers to learning; emotional barriers; discriminatory barriers; limited resources vs. positive changes Health care access challenge: availability, accessibility, affordability, adequacy & acceptability Psychosocial impact of stigma & discrimination: social interaction challenges; employment challenges; psychological impacts on emotions & self-concept Myths & superstitions: humanity & soul concerns; contagion of albinism; good & bad luck superstitions	High
Aborisade [41]	Nigeria	62	Exploratory -qualitative (low)	Semi-structured interviews (TCA)	N/A	N/A	N/A	Perceived prejudice from family in childhood; disability-specific physical violence; violence severity & impact on family relationship; coping strategies (spiritual consolation/grace of god; taking drugs; confiding in friends; mirror-talking; humor; withdrawal from others/avoidance)	High
Tambala-Kaliati et al. [32]	Malawi	27	Exploratory -qualitative (low)	In-depth semi-structured interviews; focus group (n = 2) (IPA)	N/A	N/A	N/A	Barriers to education; lack of knowledge about alb; superstitions & beliefs (alb come from mothers; alb as a family/mother curse; supernatural being); economic restraints; stigma & discrimination (humiliation; excluding society; death threats); health care challenges; personal/emotional issues; family issue (abandoned mothers; frightened families; isolating families)	High
Chu et al. [43]	Botswana	50	Cross-sectional—quantitative (moderate)	Questionnaires (OGs)	99 non-affected subjects	Presence of albinism	Health status, practices & care-access; self-perceptions; beliefs & attitude about alb	PWA: excellent understanding of the disease (90%) vs. non-PWA (63%) Most PWA felt accepted by friends (88%) and family (94%) PWA: feeling of acceptance by their community↓*; discrimination↑*; impacted by stigma in their social interactions↑ vs. non-affected subjects Almost all PWA believe they deserved extra financial and social support (95%); ½ believe albinism should be considered as a disability	Moderate
Huang et al. [42]	Taïwan	10 ♀	Exploratory -qualitative (low)	In-depth semi-structured interviews (CPM)	N/A	N/A	N/A	Discrimination; normality aspirations; sexual & maternal aspects	High
Affram et al. [44]	Ghana	105	Cross-sectional—quantitative (low)	Questionnaires (PWI-A; ISDL-SS; MSPSS)	N/A	Perceived Social support (PSS); Social stigma (SS)	Subjective well-being (SWB)	Perceived social support mediates the effect of social stigma on subjective well-being: • SS↑ indirectly → SWB↓***; • SS↑ directly → PSS from friends↓** and significant others↓**; • PSS from significant others↑ → SWB↑*	Moderate
Dapi et al. [38]	Cameroun	19	Exploratory –qualitative (low)	Focus groups (n = 3) (TCA)	N/A	N/A	N/A	Discrimination; stigma; injustice; rejection; superstition; associated skin diseases (lack of care & limited resources); knowledge toward alb + +	High
Estrada-Hernandez [31]	Puerto-Rico	8	Exploratory –qualitative (low)	Semi-structured interviews (TCA)	N/A	N/A	N/A	Knowledge about alb + + ; inappropriate social attitudes; alb boost resiliency; importance of social support; main challenges = visual impairment, sun adapt, lack of independence; need more medical awareness (HPS + +)	Moderate
Ojedokun et al. [34]	Nigeria	75	Cross-sectional—quantitative (low)	Questionnaires (ESPHWPLWA)	N/A	Social stigma (SS)	Psychological, social and health related well-being	*[Results interpretation irregularities]* SS negatively ↔ social well-being* and health well-being* (no information if these are positive/negative correlations)	Low
Christensen et al. [39]	Puerto-Rico	23	Exploratory -qualitative (low)	In-depth semi-structured interviews (CPM)	N/A	N/A	N/A	Wandering diagnosis; lack of medical consideration; lack of knowledge (uncertain future); coping = research on HPS, family support, spirituality; burden of being an expert (lead care coordination); HPS community support + +	High
Attama et al. [45]	Nigeria	100	Cross-sectional—quantitative (moderate)	Questionnaire (GHQ-28); structured interview (MINI)	100 Leprosy (lep) patients	Socio-demographic; Disease type (Leprosy vs. Albinism)	Psychiatric morbidity; psychiatric diagnosis	Psychiatric morbidity↑ for: ♂ lep vs. ♂ alb*; married lep vs. married alb*; no or low education lvl lep vs. idem alb***; sales/services & agricultural lep vs. idem alb***; self-employed lep vs. self-employed alb*** Psychiatric morbidity↑ for unmarried alb vs. unmarried lep***	High
Maia et al. [35]	Brazil	38	Cross-sectional—quantitative (moderate)	Questionnaires (WHOQOL-BREF)	40 non-affected subjects	Presence of albinism	Quality of Life (QoL)	PWA physical QoL is lower than control physical QoL***	Moderate
Phatoli et al. [33]	South Africa	5	Exploratory -qualitative (low)	Semi-structured interviews (TCA)	10 non-affected subjects	N/A	N/A	Myths & stereotypes about alb; discrimination; alb social avoidance	Moderate
Ajose et al. [36]	Nigeria	87	Cross-sectional—quantitative (moderate)	Dermatologist's clinical assessment & Questionnaire (HADS)	102 vitiligo patients	Health informations; Disease type (Albinism vs. Vitiligo)	Anxiety; Depression	Anxiety & depression↑ for: PWA with skin complications vs. without***; PWA without skin complication vs. vitiligo patients**; > 50 years old PWA vs. > 50 years old vitiligo patients** Anxiety↑ for ♀ PWA vs. ♀ vitiligo patients** Depression↓ for married PWA vs. married vitiligo patients*	High
Pooe-Monyemore et al. [40]	South Africa	15	Exploratory -qualitative (low)	In-depth semi-structured interviews (CPM)	N/A	N/A	N/A	Importance of self-concept; family role in self-concept; stigma due to appearance, myths & superstitions; role of government, non-governmental organizations, private sectors & media in development of alb recognition	High
Ezeilo [111]	Nigeria	3	Exploratory -qualitative (low)	Essay writing	N/A	N/A	N/A	Albinism as a demerit: main theme = conspicuous color, sensitive skin, visual impairments, interpersonal problems (romantic) & society's unkind attitude	Low

All study considered socio-demographic aspects (for example: age; gender; education lvl; socio-economic status; profession; etc.)

*IV* Independent variable; *DV* Dependent variable; *CPM* Colaizzi’s phenomenological approach; *TCA* Thematic content analysis; *PWA* People with albinism; *HPS* Hermansky-Pudlak syndrome; *alb* Albinism; *lep* leprosy; *lvl* level

A ↔ B: A is associated with B; A → B: A predicts B; A↑: A is higher/more important; A↓: A is lower/less important; with: w/; without: w/o; vs.: compared to…

Statistical significance index: *p* < 0.05*; *p* < 0.01**; *p* < 0.001***

#### Medical considerations

The observations are different depending on the geographical location of the studies. African studies generally report compromised access to healthcare in terms of affordability, accessibility, adequacy, availability, and acceptability [32, 37, 38, 111]. Care access is unevenly distributed over the territories, with rural areas very poorly served, unlike urban areas [32]. However, in Puerto Rico, the observations are of a different order. Firstly, in the early stages, some Hermansky-Pudlak syndrome^3^ (HPS) patients explained that it took them a long time to find out what they were suffering from due to diagnostic wandering [39]. Subsequently, participants reported that they were often discredited or not taken seriously by doctors or medical professionals [39]. In general, these PWA talk about the burden of being an expert of their own disease toward healthcare providers [31, 39], and express the need for the medical field to be more aware and more supportive of this condition [31]. This last observation inevitably leads us to discuss the relationship that PWA have with sociomedical institutions. Indeed, in some African countries there is no social assistance for PWA with very serious health conditions [38]. Furthermore, the injustices, shortcomings, and difficulties mentioned above are leading PWA to change mentalities and attitudes toward albinism. This need for recognition of albinism in society is illustrated by the fact that they have sought help from government services, non-governmental organizations [37, 38, 40], the private sector, and the media [40].

#### Educational and professional inclusion

PWA reported different shortcomings related to education and barriers to learning, including vision-related challenges, discrimination, and resource limitations [32, 37, 38]. Moreover, several studies show the extent to which visual impairments, social perceptions, and parental pressure limit academic aspirations, career choice, and employment [31, 32, 37, 38, 41].

#### Impact on family functioning

Studies showed the important role of the family in the development of self-concept (self-acceptance, self-confidence, self-esteem). First of all, the birth of a child with albinism is often experienced as an upheaval in the parents’ lives that causes fear and distress. However, family functioning drastically differs from one region of the world to another and, as for the rest of our findings, Africa gives us the most detail on these issues.

On the one hand, because of the beliefs and ritual practices that constantly threaten PWA’s lives, families are subjected to an oppressive climate of fear and stress. Parents would then tend to become overprotective of their children, a behavior that is regularly criticized by PWA once they are adults [31, 33, 38–40, 42].

On the other hand, Aborisade (2021) conducted a study on the childhood experience of PWA, whose results testified to a great propensity for intra-familial violence within Nigerian households. Between parents who abandon them and those who mistreat them, young PWA were often the target of physical violence (e.g., one third had physical scars) and psychological abuse from their relatives (e.g., taunting, name-calling, humiliation, confinement/imprisonment) [37, 42]. This violence can be attributed to guilt-ridden family relationships, especially in connection with local superstitions concerning families’ cursed blood or divine punishment. These beliefs tend to incriminate the mother as the only one responsible for the child's fate, placing guilt as the driving force in family interactions.

#### Perception of the illness and social interactions

Most studies addressed the social functioning of PWA, especially problems related to stigma and discrimination. They explained that stigma associated with albinism may be due to physical appearance (hair and skin colour) [32, 33, 37, 38, 40–43, 111] or to various myths and superstitions (“albinos don't die, they vanish”; “albinos have magical powers”; “albinism is contagious”; “albinism is the mark of a curse”; “albinos' body parts can cure diseases”; “albinos are the result of secret affairs” etc.) [32, 33, 37, 40, 42]. All of these stigmas lead society to reject and be unkind toward PWA, especially in African countries [32, 33, 37, 38, 111]. Meanwhile in Puerto-Rico, society tends to conflate the person with their impairments, giving them the feeling that they are assigned a “disabled identity” [31]. To corroborate these results, two quantitative studies have highlighted the impact of perceived social stigma on the subjective well-being of PWA [34, 44].

In this context, PWA express various difficulties and discomfort in social interactions. Firstly, many participants explained that they had been teased or bullied during childhood, especially in school [31, 32, 37, 38, 42]. Some have even faced traumatic events due to beliefs associated with their condition (aggression, violence, death threats) [32, 38, 41]. In general, studies show that PWA struggle to deal with strangers’ reactions, especially avoidance, scorn, fear, rejection, taunts, humiliation, and in the most extreme cases, aggression [31–33, 37]. Researchers also noted that PWA had difficulties in their romantic and sexual lives, including developing intimate relationships or getting married [31, 32, 37, 111]. Despite these difficulties, subjects reported the desire to reach out to others and initiate friendships [37, 40]. In Puerto Rico for example, the author showed that PWA interacted very well with their peers and made strong friendships [31]. By contrast, some authors also reported that some PWA, overwhelmed by social pressure, preferred to withdraw from social situations to avoid being noticed [41, 111]. Furthermore, the studies showed that social support is the most cited coping strategy of PWA. They identified the importance of having family members, close friends, neighbors, and teachers to rely on, and that made them feel included [31, 33, 37–39, 41, 111]. To support this idea, Affram and his colleagues (2019) showed that social support from significant others partially mediated the effect of social stigma on subjective well-being. Lastly, the studies led in Puerto Rico showed that the participants (mostly Hermansky-Pudlak syndrome) identified the patient’s community as a great source of support, a space where they could share their experiences and feel understood [31, 39].

#### Impact on physical functioning

Several studies showed the impact of albinism on physical functioning, particularly for visual impairment and skin vulnerable to the sun [31, 32, 37, 38, 111]. To reinforce these findings, a study conducted in Brazil showed that the physical dimension of WHOQOL (World Health Organization Quality of Life) was negatively impacted in PWA [35]. A study conducted in Puerto Rico on subjects with HPS also reported the impact of such a disorder on the circulatory and respiratory systems (e.g., coagulopathy; and in some cases, neutropenia, or pulmonary fibrosis) [39].

#### Impact on psychological functioning

Finally, at an individual level, participants' perceptions of their disease reflect a good understanding of albinism, which they define as a genetic and inherited condition [31, 33, 36, 43]. However, perceptions of albinism seem to differ according to where the studies were carried out. African PWA were indeed more likely to perceive albinism as a curse [32, 33, 41], while in Puerto-Rico, PWA perceived it more as a source of resilience, an opportunity for self-improvement [31]. Secondly, several studies revealed a major complex that PWA have regarding normality. While some expressed willingness to be seen as normal [33, 42], others reported that they were under social pressure related to the desire to fit society's norms (particularly in relation to performance) [31]. To overcome this “normality complex” problem, some studies emphasized the inexhaustible strength of self-acceptance: a state of mind that develops through education provided by parents [40], or through therapeutic courses [42]. Nevertheless, the symptoms of albinism still have an impact on daily life. For instance, Estrada-Hernandez (2018) highlighted the frustration of PWA not being able to be completely independent in terms of mobility; for example, because of their visual problems, PWA cannot drive a car. The hereditary nature of albinism also raises an important issue: the guilt about the risk of passing on their albinism to their children. Many mothers with albinism have been faced with this dilemma and have had to make choices (i.e. make the decision not to have children, to have a termination of pregnancy, etc.) [42]. Finally, the study conducted among HPS patients reports their uncertainty about the future due to the unpredictability of the disease's evolution [39]. All in all, PWA have learned to develop a wide variety of coping strategies (spiritual consolation/complaining to God, scientific knowledge seeking, social support, humor etc.) [39, 41, 111] related to the development of a “fighting spirit” [31].

In view of all the above, we can detail several psychological consequences observed in these studies, first at the emotional level: sadness, fear, anxiety, anger, resentment, shame, feelings of hopelessness, powerlessness, helplessness, worthlessness, bitterness, loneliness, and frustration [32, 37, 38, 41]; and in terms of thought and behavior patterns: low self-esteem, low self-confidence, self-isolation, low self-efficacy, distrust of others, paranoia, avoidance, denial, guilt, and self-harm or self-reproach [33, 37, 38, 40, 41, 111]. Lastly, a study in Nigeria showed that PWA with severe skin complications tended to be more anxious and depressed than those without. Their results also showed that PWA with severe skin problems were as anxious and depressed as subjects with vitiligo [36].

### *Research on NF1: n* = *16*

A total of 1180 participants were included across NF1-related studies. Most studies have been conducted in Europe (Norway, *n* = 2; England, *n* = 2; Germany, *n* = 1; Italy, *n* = 1; France, *n* = 1; Sweden, *n* = 1) and in the USA (*n* = 5). The other research was led in Australia (*n* = 1), Brazil (*n* = 1) and Iran (*n* = 1). Eighty-one percent of the studies included mostly female participants (6% with female-only participants, 75% with more than half female participants). The sex ratio was balanced in only two qualitative studies [112, 113], with men outnumbering women in only one study [114]. Overall, women represented 66.22% of the participants in all the samples. Among the studies we identified, four of them also included participants under 18 years old [115–118] (see Table 3 for more details).

**Table 3:** Details of reviewed studies about neurofibromatosis type 1, according to location, sample, method design and significant outcomes

Setting			Method					Results	Study quality
Ref.	Location	N	Design (strength)	Tools	Comparator(s)	IV(s)	DV(s)	Significant outcomes	
Foji et al. [121]	Iran	24	Exploratory -qualitative (low)	Interview w/ evolving structure (GTM)	N/A	N/A	N/A	NF1 patients’ life & their response to failure & falling behind in life $\approx$ ‘an unsuccessful struggle to escape’ Environmental conditions: unpleasant appearance due to spots & tumors; inability to have kids; learning disabilities; limitations daily life activities; social rejection and isolation; facing aggression form others; perception of no social support; incurability of NF1 Indivuals’ responses/coping: hiding disease from others; seeking isolation; complaining to God; refusing to receive care; hopelessness; In extreme cases: suicidal ideation, unsuccessful suicide attempts	High
Fjermestad et al. [123]	Norway	142	Cross-sectional—quantitative (low)	Questionnaires (HUNT3; OGs)	46,393 non-affected subjects (extracted from a cohort study)	Biomedical datas; Mental functioning; Health related QoL	Life satisfaction	HrQoL problems↑ in following domains: life satisfaction, mental health, sleep, pain, gastrointestnal problems, oral health, memory problems, social support (especially women) MLR model → life satisfaction: mental health, sleep, pain, memory problems, social support*** Only mental health was a unique significant predictor***	Moderate
Jensen et al. [115]	USA	16	Exploratory -qualitative (low)	Semi-structured interviews (TCA)	N/A	N/A	N/A	Chronic pain & acute episodes of localized pain; social functioning (limited activity participation, role limitation & relationship impact); mobility difficulties; internalized stigma (more external in youth)	Moderate
Rosnau et al. [116]	USA	49	Cross-sectional—quantitative (low)	Questionnaires (RSES; OGs)	General population norms (Sinclair et al. 2010)	Biomedical datas; NF1 experiences	Self-esteem (SE); NF1 knowledge	¾ of the participants had a quite good knowledge about NF1 NF1 SE↓ vs. general pop. norms*** MLR model → SE: learning problems, having friends with NF1, attending a support group & receiving genetic counseling*	High
Bicudo et al. [119]	Brazil	13	Cross-sectional mixed—quali-quantitative (moderate)	Hetero-assessment scale (Ablon & Riccardi); questionnaires (WHOQOL-100) & Semi-structured interviews (TCA)	39 non-affected subjects	NF1 severity & visibility	Quality of Life	No significant impact of NF1 on QoL Difficulties: pain; concern about the future; shame; discomfort; awkwardness; hiding body; concern about genetic counseling; limited job opportunity & professional life; NF1 as a curiosity for strangers; confusion from strangers with contagious diseases; social prejudices; inappropriate healthcare; lack of information Coping: spirituality/religion/beliefs; relativizing NF1; social support (e.g. family); resection of pNF	Moderate
Crawford et al. [122]	Australia	60	Exploratory -qualitative (low)	Interview w/ evolving structure (GTM)	N/A	N/A	N/A	Cosmetic disfigurement as a burden; social discomfort & awkwardness; difficulty in finding partners; lack of awareness and knowledge of NF1 in society; learning and/or attention difficulties in childhood; affected aspirations & self-esteem; genetic inheritance concerns; unpredictable disease progression; pain	High
Barke et al. [117]	England	9	Exploratory -qualitative (low)	Semi-structured interviews (TCA)	N/A	N/A	N/A	NF1 impacts differs a lot (severity & visibility); social discomfort & awkwardness; social support (family, friends, online support group); adolescence as a period of learning & awareness about NF1; NF1 poorly understood (medical community, medias); unpredictable disease progression	Moderate
Hummelvoll & Antonsen [120]	Norway	15	Exploratory -qualitative (low)	Semi-structured interviews (TCA +)	N/A	N/A	N/A	Pain (sleep disturbance, fatigue); movement & mobility difficulties; anxio-depressive symptoms; the importance of family background and relations; the role of friendships; low self-confidence; dealing with NF1 visibility; unpredictable disease progression; personal/cognitive aspects mediates the impact of NF1	High
Smith et al. [126]	USA	127 ♀	Cross-sectional—quantitative (moderate)	Questionnaires (DAS59; RSES; UCLA Loneliness Scale)	48 NF2 ♀ + General population & Breast Cancer Survivors’ (BCS) norms (extracted from other studies)	Appearance distress / self-consciousness	Self-esteem (SE); Loneliness	NF1 women reported more psychosocial reasons for disliking their feature vs. NF2 women* NF1 sexual / bodily self-consciousness↑ vs. general pop norms* and BCS*** NF1 social self-consciousness↑ vs. general pop norms and BCS*** NF1 sexual / bodily self-consciousness↑ ↔ SE↓*** NF1 social self-consciousness of appearance↑ ↔ SE↓***; loneliness↑**	High
Granström et al. [127]	Germany	228	Cross-sectional—quantitative (low)	Hetero-assessment scale (Riccardi); Questionnaires (DLQI; Distress Thermometer; FBeK; OGs)	2047 non-affected subjects + 105 psoriasis subjects (extracted from other studies)	Biomedical datas; NF1 severity & visibility; Body Image	Depressive statement; Psychological distress; Health-related QoL	NF1 visibility↑ ↔ depressive state↑**, frequency of a lifetime depression diagnosis↑**, psychosocial distress↑***, QoL impairment↑*** and body experience↓*** NF1 patients’ feelings: insecure and uneasy with their own bodies↑***, attractive and self-confident↓*** vs. healthy pop NF1 visibility effect on depressive state was completely mediated by NF1 patients’ body experience***; partially on psychological distress*** and QoL impairment**	High
Dheensa & Williams [112]	England	6	Exploratory -qualitative (low)	In-depth semi-structured interviews (IPA)	N/A	N/A	N/A	Lack of information about NF1 (no further explanations; loss faith & trust in medical professions); feeling judged (social self-consciousness aroused; isolation/solitude); social comparisons; variety of coping methods; unpredictable disease progression; some positive appraisal	Low
Kodra et al. [125]	Italia	129	Cross-sectional—quantitative (low)	Hetero-assessment scale (Ablon) & Questionnaires (SF-36; Skindex-29)	N/A	NF1 visibility	General & skin-disease-specific QoL	NF1♀ impact on emotions↑** and physical↑*** symptoms vs. for NF1♂ NF1 visibility↑ independently ↔ skin-disease-specific QoL following aspects: emotions↓***, physical symptoms↓* and functioning↓***	Moderate
Page et al. [124]	USA	169	Cross-sectional—quantitative (low)	Hetero-assessment scale (Ablon & Riccardi); Questionnaires (SF-36; Skindex-29)	154 non-affected U.S. subjects (extracted from another study)	NF1 severity & visibility	General & skin-disease-specific QoL	NF1 visibility↑ independently → skin-disease-specific QoL following aspects: emotions↓ (♀ especially)**, symptoms↓* & functioning↓*** NF1 severity↑ independently → skin-disease-specific QoL following aspects: symptoms↓*, function↓* NF1 patients general health QoL↓ vs. normative pop* NF1 severity↑ independently → general health QoL↓ (physical function, bodily pain, general health perception, vitality, role emotional, mental health, social functioning, role physical, physical symptoms)**	Moderate
Wolkenstein et al. [118]	France	128	Cross-sectional—quantitative (low)	Hetero-assessment scale (Ablon & Riccardi); Questionnaires (SF-36; Skindex-29)	3656 subjects representative of the French population (extracted from another study)	NF1 severity & visibility	General & skin-disease-specific QoL	NF1 impact on emotions↑*** & physical symptoms↑** for ♀ vs. ♂ NF1 visibility↑ independently → skin-disease-specific QoL following aspects: emotions↓*, symptoms↓** & functioning↓** Each aspect of NF1 general health QoL↓ vs. normative pop NF1 severity independently → 4 aspects of general health QoL: physical function**, bodily pain*, general health perception**, vitality* NF1 visibility independently → 4 aspects of general health QoL: physical function**, social functioning*, role-emotional*, mental health*	Moderate
Zöller & Rembeck [114]	Sweden	70 (1978) → 37 (1990)	Longitudinal study—quantitative (12 years follow-up) (moderate)	Medical & psychiatric interviews (CPRS); Questionnaires (KSP; SES)	27 non-affected subjects	NF1 severity (quantity & location of neurofibromas)	Psychiatric characteristics; Personality profile; Self-concept	NF1 patients: mood disorders↑ (depression, dysthymia) vs. non-affected subjects*** NF1 patients: sleep↓*; social phobias↑*; worries about trifles↑** vs. non-affected subjects NF1 patients w/o psychiatric diagnosis: self-evaluation & socialization↑*; aggressivity↓***; irritability↓** vs. non-affected subjects No further difference between 1978 & 1990	Moderate
Ablon [113]	USA	28	Exploratory -qualitative (low)	One question-based interviews	N/A	Gender	Life experiences	♀ ≠ ♂: internalized cosmetic norms & body concerns; parenting prevail over gender; fears of rejection harder for ♀: genetic inheritance concerns; more binding appearance norms harder for ♂: compromised manliness; employability & professional life; represses their feelings; tend to withdraw from social life & avoid romantic situations (unlike women)	Low

All study considered socio-demographic aspects (for example: age; gender; education lvl; socio-economic status; profession; etc.)

*IV* Independent variable; *DV* Dependent variable; *GTM* Grounded theory methodology; *TCA* Thematic content analysis; *NF1* Neurofibromatosis type 1; *MLR* Multiple linear regression; *OGs* Originals instruments; *pop* population

A ↔ B: A is associated with B; A → B: A predicts B; A↑: A is higher/more important; A↓: A is lower/less important; with: w/; without: w/o; vs.: compared to…

*p* < 0.05*; *p* < 0.01**; *p* < 0.001***

Concerning the quantitative results, eight studies were conducted involving 1009 participants (m = 126.13). The average age of participants varied from 37 to 51 years (m = 42.65, *SD* = 4.93). Almost all cross-sectional studies used a control strategy by extracting data from other studies (*n* = 6) or setting up a group of non-affected subjects (*n* = 2). Concerning the qualitative results, seven studies have been considered, involving 158 participants (m = 22.57). Only one mixed-method study was incorporated and included 13 participants [119].

#### Medical considerations

The studies reported a general lack of information about NF1 [112, 119] and NF1 appears to be poorly understood in the medical community, particularly by general practitioners [117]. In these circumstances, the available healthcare is not always appropriate, and the diagnosis is rarely followed by sufficient information and personalized support and counseling that would allow a concrete explanation of what NF1 is [112, 119]. In order to cope with the fear of tumor development, patients are regularly followed-up by a specialist [120]. It should also be noted that resection of neurofibromas is a real health need for many patients [119]. To corroborate these findings, Rosneau et al. (2017) found that subjects’ self-esteem was higher if they received care at a NF clinic or had received genetic counseling.

#### Impact on family functioning

Family functioning is also greatly impacted by NF1 as it brings worry or guilt about the partner, the children, or future children. NF1 is a “family affair”; considering its inherited genetic nature, NF1 is often a well-known genealogical issue [117]. This familial issue generates a “hereditary anxiety” mentioned in many studies and which is characterized by the fear or guilt that NF1 could be passed to children [115, 119–122]. This fear is the reason why some mothers have decided to never have children [121, 122]. To this extent, many couples have resorted to prenatal tests to assess the risk of the disease’s transferability [121]. Other women, on the other hand, did not feel bound by heredity. However, the birth of an NF1 baby is often experienced as a great upheaval in the family [117] and causes many concerns about the future and the development of the child's NF1 [119, 120]. These latter observations highlight the importance of knowing the genetic background of the family. Indeed, diagnosis in a family without NF1 leads to a lot of misunderstandings, diagnostic wandering, and can be source of conflicts within the family [120].

#### Perception of the illness and social interactions

NF1 significantly alters social functioning. Several studies showed that many patients have experienced stigma because of their physical appearance, in particular the presence of plexiform neurofibroma (PNf) [115]. Many participants identified adolescence as a time when stigma emerged as a prominent concern [115], particularly because they could be the victim of teasing or bullying [121, 122]. In this context, NF1 patients expressed several difficulties and discomfort in social interactions and several studies showed that participants had difficulties dealing with strangers’ reactions [115, 117, 119, 121, 122]. In most social situations, NF1 patients are seen as a curiosity [115, 117, 119] and in the most extreme cases, they may have been rejected, humiliated, even experienced aggression [121, 122]. In addition, some studies showed that some patients had difficulties forming romantic relationships despite a desire to do so [115, 122]. This kind of difficulty was illustrated in one study by the apprehension that some patients felt about having sexual relations [122]. Moreover, in Iran, researchers have shown that NF1 is a real barrier to marriage [121]. However, there does not seem to be a consensus among these observations, as another study indicated very precisely that NF1 had never been an obstacle to the development of romantic relationships [119]. Considering all these elements, participants deplored being victims of social prejudice and being treated differently than others, even sometimes treated as stupid [112, 119, 121]. In this context, some authors reported that some NF1 patients had a diminished interest in participating in social activities [112, 115, 121]. To compensate for these difficulties, participants knew how to exclusively seek help from their family, close friends, or friends from NF1 patient organizations [117, 119, 120]. To support this point, Rosneau et al. (2017) found that subjects’ self-esteem was higher if they had friends with NF1 or attended an NF1 support group. Nevertheless, some participants preferred online support rather than face-to-face groups [117].

These difficulties can be partially explained by social perceptions of the illness. It should also be noted that, once again, there is a consequent lack of awareness and knowledge about NF1 [119, 122]. In addition, the media convey stereotypes that give a negative and pathologizing image of NF1 (e.g., “Elephant man” disease) [117]. A study also reported some misconceptions that people had about NF1, especially that it was a contagious disease [119]. As a result, to compensate for this lack of knowledge, many patients took the initiative to inform the people around them about NF1 [120].

#### Impact on physical functioning

Firstly, one of the most important symptoms of NF1, but which was not expressed by all patients, was the presence of pain (acute or chronic) and/or uncomfortable body sensation [115, 119, 120]. This physical sensation is usually caused by the development of PNf in patients’ skin. In addition, those who experienced chronic or severe pain were negatively and significantly impacted in health and daily functioning [122]. Other authors showed that pain disturbed sleep and increased fatigue [114, 120, 123]. Some studies also found that NF1 caused movement and mobility impairments (skeletal disorders and tumours, reduced ability to walk, dizziness, vision problems, and sound or photosensitivity) [115, 120, 121]. In this way, several authors showed that the severity of NF1 was especially associated with an impaired QoL [118, 124]. In contrast, one quantitative study found no effect of NF1 on QoL, either in terms of severity or of visibility (Bicudo et al. 2016). This surprising result may be due to the small sample size of this research (*n* = 13).

#### Impact on psychological functioning

Firstly, cross-sectional studies showed that NF1 has a negative impact on QoL [118, 123–125], self-esteem [116, 126] and body image (appearance-related concerns) [126, 127]. Many patients also reported that NF1 affected their cognitive abilities during childhood, particularly in terms of learning and attention [117, 120, 122]. Cosmetic disfigurement was globally experienced as a burden that causes emotional distress such as shame [119, 120, 122], discomfort, awkwardness [119], self-consciousness, low self-confidence [122], or self-contempt [120]. NF1 visibility was also associated with an altered QoL [118, 124, 125, 127], depressive state, psychosocial distress, and negative body experience [127]. Smith and colleagues (2013) showed that NF1 patients' self-esteem could be negatively predicted by the degree of awareness they may have regarding their own appearance. To overcome this difficulty, many participants explained that they hide body parts that are particularly affected by NF1 [119–122]. In addition, NF1 seems to affect their autonomy, making them less independent in their personal life [119, 121]. As a result, perceived stigma and altered autonomy impact vocational decision-making and discourage people with NF1 from pursuing their desired professions [115, 121, 122].

The unpredictable evolution of NF1 appeared to be one of the most stressful aspects of the disease (appearance of new pNF or other tumors for example) [112, 117, 119–122]. In light of all these elements, the studies showed that all participants developed a wide range of coping strategies [112, 121]. Among them, we can find two opposing states of mind that act as a “cognitive-coping-posture”: physical hypervigilance vs. existential relativism [120]. The latter can be illustrated as state of acceptance and an awareness of NF1 as an incurable life-long disorder, i.e., as a part of them that cannot be changed [122]. Otherwise, some participants relate to spirituality, religion, or other personal beliefs to help them live with their condition and associated symptoms [119, 121]. For others, seeking information and gaining knowledge about NF1 seemed to be essential in their disease management. In their teenage years, some patients sought to learn more about NF1, to be more aware of it, and became experts on their own condition [117].

To summarize, the experience of NF1 can affect subjects’ mental health. For example, Crawford and his colleagues showed that pain was associated with anxiety, stress, low mood, and depressive symptoms. Thus, subjects have a propensity to develop mood disorders and anxio-depressive symptoms [114, 123], and to have low self-confidence [120] and low self-esteem [122]. Finally, two studies showed that NF1 had more impact on women, particularly on their emotional experiences and physical functioning [118, 124].

### *Research on inherited ichthyosis: n* = *10*

A total of 1228 participants were included across ichthyosis-related studies. Most of the studies identified were conducted in Europe (*n* = 7), especially in France (*n* = 3) and Sweden (*n* = 2). Two other studies were carried out in the U.S. More recent studies have been conducted in Wales (*n* = 1), Italy (*n* = 1) and Mexico (*n* = 1). The proportion of adult male and female participants cannot be reported as it is not precisely indicated in two studies [90, 91], but overall, in every study, women are more represented than men. In addition, five studies included participants under 18 years of age, making it difficult to calculate the mean age of the adults included in these 10 studies. Approximately 230 subjects under 18 years old were considered in these samples. Eight studies relied on a quantitative cross-sectional design and gathered 1193 subjects while the two remaining studies used either an exploratory qualitative method [92] or a mixed design [128] (Table 4).

**Table 4:** Details of reviewed studies about ichthyosis according to location, sample, method design and significant outcomes

Setting			Method					Results	Study quality
Ref.	Location	N	Design	Tools	Comparator(s)	IV(s)	DV(s)	Significant outcomes	
Wren et al. [95]	Wales	371 (54 XLI♂; 83 ♀XLI-carrier; 82 IV; 152 psoriasis)	Cross-sectional—quantitative (moderate)	Questionnaires (CISI, PASI, K10; ASRS; AQ10; BITE; DLQI; FSQ)	1116–18,700 non-affected subjects, students or outpatients (extractions from other studies)	Condition group; skin disease severity	Mood/neurodevelopmental traits; Skin-disease-specific QoL; stigmatization	All groups: psychological distress↑ (i.e. depression↑; anxiety↑) vs. non-affected subjects*** All groups: atypical neurodevelopmental trait↑ (i.e. ASD↑; ADHD↑) vs. non-affected subjects* IV ♀: ichthyosis severity↑ ↔ recent adverse mood symptoms↑* Main factors influencing XLI & IV mood: stigma / bullying; embarrassment of social situation; reduced social life (esp.♂-depression); difficulty regulating body temperature (esp.♀-irritability); skin related discomfort & difficulties w/ treating ichthyosis (esp.♀-irritability & ♂-anxiety)	Moderate
Abeni et al. [94]	Italy	94 (including 52 subjects < 18 yrs)	Cross-sectional—quantitative (low)	Questionnaires (DLQI; FBI)	N/A	Biomedical datas; Ichthyosis severity & symptoms	Skin-disease-specific QoL; Family burden	QoL↓ ↔ Disease severity↑* (e.g. fissures*, itch**, recurrent infections**, walking problems**) QoL aspects impacted = itch & pain*, embarrassment & self-consciousness*, problems w/ clothing choice*, problems caused by treatment* Family burden↑ ↔ Disease severity↑* (e.g. thick scales*, fissures*, or foul smell*) + harlequin & lamellar ichthyosis** Psychological dimension of Family burden were the most impacted*	Moderate
Cortès et al. [97]	Mexico	26 (only LI)	Cross-sectional—quantitative (moderate)	Questionnaires (CISI; DBI-II; DLQI)	26 non-affected subjects	Ichthyosis severity	Depression; Skin-disease-specific QoL	LI patients’ depression↑ vs. non-affected subjects*** No correlations between ichthyosis severity ↔ depression / QoL	Moderate
Sun et al. [93]	USA	181 (including 53 subjects < 17 yrs)	Cross-sectional—quantitative (low)	Questionnaires (DLQI; PHQ-9; GAD-7)	N/A	Biomedical datas (e.g. ichthyosis features)	Skin-disease-specific QoL; Depression; Anxiety	Adults w/ ichthyosis: QoL impairments (95%); depression + (34%); anxiety + (27%) Adults w/ ichthyosis: Leisure impairment ↔ depression↑***; anxiety↑* Adults w/ ichthyosis: Difficulties at work ↔ anxiety↑*	Moderate
Dreyfus et al. [90]	France	241 (including 70 subjects < 18 yrs)	Cross-sectional—quantitative (low)	Questionnaires (VAS; DLQI)	N/A	Medical care; out-of-pocket expenses; work/school/leisure activities; Ichthyosis severity	Skin-disease-specific QoL	Medical care: regularly followed by a physician (90%); no feedback about genetic tests (70%) Impact on domestic life: moisturizing creams each day (94%); affects clothing & footwear (71%); additional housework (47%); negative impact on familial and conjugal functioning (25%) Financial burden: care expenses not fully covered (48%); out-of-pocket expenditure (86%)$\approx$ 526€/yr Impairment in outside activities: workplace discrimination (27%), restriction in leisure & sports (e.g. swimming-pool) (35%)	Moderate
Dreyfus et al. [91]	France	158 (including about 20 subjects < 18 yrs)	Cross-sectional—quantitative (low)	Questionnaires (VAS; DLQI)	N/A	Ichthyosis severity	Skin-disease-specific QoL	The most affected QoL areas: symptoms & feelings (86%); daily activities (77%); treatments (62%); work (59%); leisure (55%); personal relationships (45%) QoL↓ ↔ Ichthyosis severity↑*** & ♀*	Moderate
Mazereeuw-Hautier et al. [92]	France	25	Exploratory -qualitative (low)	Focus groups (n = 5) (TCA)	N/A	N/A	N/A	Physical health: pain & impaired mobility, pruritus, smelly skin Daily life: activity or work avoidance, daily cream application Relation to others: dealing w/ other reactions, intimate relations issues, lack of interest from medicine Personal aspects: fear / stress related to future, negative feelings	High
Kamalpour et al. [96]	USA	235 (including about 70 subjects < 18 yrs)	Cross-sectional—quantitative (low)	Questionnaires (DLQI; CISI; TimeTx; DermVisits)	N/A	Biomedical datas; Ichthyosis severity	Skin-disease-specific QoL; Resource utilization	Adults QoL↓ vs. children* + ♀ QoL↓ vs. ♂* QoL↓ ← erythema severity↑**, hyperkeratosis severity↑**, time spent in daily treatment↑**, ichthyosis type**, age↑* *(the last one’s really weak…)* TimeTx↑ & DermVisits↑ ← disease severity↑*, age↑* (+ family history → DermVisits*)	Moderate
Gånemo et al. [129]	Sweden	122	Cross-sectional—quantitative (moderate)	Questionnaires (DLQI; SF-36)	117 non-affected subjects (extracted from another study)	Biomedical datas (e.g. ichthyosis features)	General & skin-disease-specific QoL	LI patients QoL↓ vs. XLI patients* Ichthyosis patients QoL↓ on 4 dimensions (…) vs. normative pop.*	High
Gånemo et al. [128]	Sweden	10	Cross-sectional mixed—quali-quantitative (low)	Semi-structured interviews (TCA) & questionnaires (NHP-I & II)	N/A	Biomedical datas; Ichtyosis severity	Health-related QoL; Health-related problem in daily life	Childhood: parents deeply engaged; feeling different from peers; bullying; shyness; gynophobia; professional orientation; thoughts of how to find a partner Adulthood: regular doctor appointment (or naturopath, homeopath); improvement of symptoms; time-consuming skin care; skin discomfort (heat intolerance); genetic inheritance concerns	Moderate

All study considered socio-demographic aspects (for example: age; gender; education lvl; socio-economic status; profession; etc.)

*IV* Independent variable; *DV* Dependent variable; *GTM* Grounded theory methodology; *TCA* Thematic content analysis; *XLI* X-linked ichthyosis; *IV* Ichthyosis vulgaris; *LI* Lamellar ichthyosis; *HI* Harlequin ichthyosis; *MLR* Multiple linear regression; *ASD* Autism spectrum disorders; *ADHD* Attention-deficit/hyperactivity disorder; *pop* population

A ↔ B: A is associated with B; A → B: A predicts B; A ← B: A is predicted by B; A↑: A is higher/more important; A↓: A is lower/less important; with: w/; without: w/o; vs.: compared to…

*p* < 0.05*; *p* < 0.01**; *p* < 0.001***

#### Medical considerations

This disease requires regular follow-up by doctors [128]. Unfortunately, the medical world does not seem to have much interest in patients with ichthyosis [92].

#### Educational and professional inclusion

Many subjects explained the constraints they have encountered in their professional orientation [128] and shared the difficulties they had at their workplace [90–92]. These difficulties were associated with a higher degree of anxiety [93].

Studies also reported that this skin condition restricted them in certain outdoor activities, such as playing sports or other leisure activities [90, 91, 128].

#### Impact on family functioning

Some researchers have highlighted the impact of ichthyosis on family functioning. Disease severity was positively associated with family burden, and some types of ichthyoses (associated with more severe symptomatology) were also related to a greater family burden, especially harlequin and lamellar ichthyosis [94, 129]. Here again, the hereditary nature of the disease seems to be a central issue in family functioning [128].

#### Perception of the illness and social interactions

Many participants reported that they had been teased or bullied by some of their peers in their childhood and adolescence [95, 128]. As they moved into adulthood, several participants expressed discomfort and embarrassment during social interactions, especially when others pointed out or questioned them about their skin lesions [92, 95]. In addition, the skin condition can cause problems in intimate relationships [92] thus impacting the conjugal life of some patients [90].

#### Impact on physical functioning

We can already note that ichthyosis alters physical functioning. Indeed, ichthyosis causes pain and/or uncomfortable body sensations such as damaged skin (e.g., fissures, itching, pruritus, infections), body odour, or heat intolerance [92, 94, 128]. In addition to this, participants explained that physical symptoms could increase over time [128].

#### Impact on psychological functioning

Quantitative studies show that ichthyosis has a negative impact on QoL [91, 93, 94, 96, 129] and caused anxiety and depressive states in patients [95, 97].

In those articles, the most studied predictor was self-perceived ichthyosis severity, which was associated with a higher negative impact on QoL [90, 91, 94, 96] and recent adverse mood symptoms [95]. However, Cortès and colleagues found no predictive link between the severity of ichthyosis and QoL. Furthermore, adults seem to have greater QoL impairments due to ichthyosis than children [96].

Some authors have recently identified the main factors influencing mood (in terms of anxiety, depression, and irritability). We can mention for example stigma, bullying, embarrassment in social situations, reduced social life, body hyperthermy, skin discomfort and treatment [95].

This brings us to discuss the impact of ichthyosis on daily life. Subjects reported that they should apply moisturizing cream very regularly to limit skin complications [90–92, 128] but skin care visibly seems to be time consuming for some participants [95], and time spent in daily treatment seems to be associated with a greater impact on QoL [96]. Additionally, ichthyosis symptoms have an impact on participants' mobility (e.g., walking problems), sleep, and clothing [90, 92, 128], making it difficult to do certain activities. Thus, researchers have observed that impairments in leisure activities were associated with a higher anxio-depressive state [93]. Finally, Mazereeuw-Hautier and colleagues (2012) highlighted the stress linked to the unpredictable nature of the disease and its evolution [92].

### *Research on birthmarks: n* = *6*

A total of 657 participants with birthmarks were included in the five identified studies. Three studies were led in Europe (*n* = 3), one in China, another in Australia, and the oldest one in the U.S. Overall, women were more represented than men (70.17%). The mean age could not be calculated because this information was missing in two articles [102, 130], and two studies included some adolescents in their samples [130, 131]. Five studies were based on a quantitative cross-sectional design and included 634 PWS subjects [101, 102, 130–132]. Almost all these studies used a comparison-control strategy, except for one [130]. The sixth study, which is also the oldest, was built on a qualitative exploratory design and included 23 subjects [133]. The only qualitative study showed that several dimensions of the participants' lives were affected by their birthmarks (Table 5).

**Table 5:** Details of reviewed studies about birthmarks, according to location, sample, method design and significant outcomes

Setting			Method					Results	Study quality
Ref	Location	N	Design	Tools	Comparator(s)	IV(s)	DV(s)	Significant outcomes	
Wang et al. [102]	China	197	Cross-sectional—quantitative (moderate)	Questionnaire (DLQI)	196 vitiligo subjects	PWS characteristics	Skin-disease-specific QoL	PWS patients QoL↓ vs. vitiligo patients (esp. feelings, daily activities, leisure, work/school, treatment)*** QoL↓ ← ♀; hypertrophy↑; size of skin lesion↑*	High
Hagen et al. [101]	USA	244	Cross-sectional—quantitative (low)	Questionnaire (Skindex-29; OGs)	14 other skin conditions extracted from previous studies	Socialization w/ others; medical comorbidities; PWS severity; treatments	Skin-disease-specific QoL	Anxiety & depression: most reported comorbidities and associated w/ impact on QoL↑*** QoL ↔ comorbid depression**, limited facial mobility**, presence of other skin conditions* Emotional impairments↓ ↔ older patients**, patients from educationnal services*** PWS hypertrophy ↔ emotional↑* & symptomatic↑*** impairments Functional impairments↓ ↔ close friends↑* & social engagements↑* PWS patients: QoL↓ vs. non-affected subjects (but similar to CTCL, rosacea, alopecia & vitiligo)	Moderate
Augustin et al. [132]	Germany	70	Cross-sectional—quantitative (low)	Dermatologist's clinical assessment; Questionnaires (SCL-53R; ALLTAG; CSDQ; FKS; OGs)	1006 non-affected subjects (extracted from another study)	Skin-specific coping; impact of PWS; global QoL limitations	Emotional well-being; body image	PWS patients: emotional well-being↓ (interpersonal sensitivity***, anxiety**, hostility**, phobic anxiety**, paranoid ideation**); body perception↓ (attractiveness/self-confidence*); QoL↓ (social relationship*) vs. non-affected subjects Body perception & emotional well-being ↔ physical malaise*	High
Ben-Tovim & Walker, [131]	Australia	52 (including about 5 adolescents)	Cross-sectional—quantitative (moderate)	Questionnaires (BAQ)	49 rheumatoid arthritis; 23 eczema/psoriasis; 50 type 1 diabetes; 174 non-affected subjects	Body informations (height, weight, BMI)	Body-related attitudes	BVD patients: body-related attitudes impacted (strength/fitness↑*; salience of weight/shape↓*)	Moderate
Lanigan & Cotterill, [130]	England	71 (including about 5 adolescents)	Cross-sectional—quantitative (low)	Questionnaires (GHQ; HADS; OGs)	N/A	PWS characteristics	General health; Emotional state; Attitudes toward their PWS	No significant impact of PWS on emotional or psychiatric state Social difficulties in PWS patients: dealing with strangers' reactions; need to hide the mark or treat it; impact on self-confidence; feeling different from others; feeling unattractive	Moderate
Malm & Carlberg, [133]	Sweden	23	Exploratory -qualitative (low)	Structured interviews	N/A	N/A	N/A	Patients with Large PWS: dealing with strangers' reactions (emotionally difficult); family as a source of support; childhood's bad experiences; very different coping in social contexts (withdrawall, taunt, humor); mark-hiding techniques (make-up) Patients with Small PWS: mark-hiding techniques (make-up); dealing with strangers' (paranoid anxiety about PWS); exaggerated self-consciousness about the PWS	Low

All study considered socio-demographic aspects (for example: age; gender; education lvl; socio-economic status; profession; etc.)

*IV* Independent variable; *DV* Dependent variable; *GTM* Grounded theory methodology; *TCA* Thematic content analysis; *PWS* Port-wine-stains; *CTCL* Cutaneous T-cell lymphoma; *BVD* Blood vessel disorders; *MLR* Multiple linear regression; *pop* Population

A ↔ B: A is associated with B; A → B: A predicts B; A↑: A is higher/more important; A↓: A is lower/less important; with: w/; without: w/o; vs.: compared to…

*p* < 0.05*; *p* < 0.01**; *p* < 0.001***

#### Social interactions and perception of birthmarks

Many participants reported having difficulties in dealing with people's reactions [130, 133]. It should also be noted that the study conducted by Malm and Carlberg (1988) focused on the differences in the experiences of people with large PWS and small PWS. Participants with large PWS reported having negative childhood experiences with some of their peers [133], making them feel different from other children [130, 132]. In order to overcome these problems, those with birthmarks developed very different ways of coping in social contexts, such as taunts, humor, and avoidance or withdrawal [132, 133]. In addition, subjects identified family and friends as a great source of support [133].

#### Impact on psychological functioning

All the elements mentioned above have repercussions on the individual and psychological functioning of people with birthmarks. Firstly, these studies showed that, compared to non-affected people, patients with birthmarks have an altered QoL [101, 132], lower emotional well-being [132] and an impaired body image [131, 132]. Hagen and colleagues (2017) observed that the more PWS is severe (tissue hypertrophy, birthmark size), the greater the negative impact on QoL, especially in emotional and symptoms-related domains. However, this result is not consensual, as Lanigan & Cotterill (1989) didn’t find any significant impact of PWS on emotional or psychiatric state. Regarding body image, one study showed that feelings of attractiveness and body-related self-confidence were impacted [132]. Furthermore, body-related attitudes were altered in people with birthmarks, with an acute vigilance to body strength, weight, and shape compared to unaffected people [131]. As a result, studies reported a lack of confidence in people with birthmarks and the fact that they felt unattractive [130, 132]. To that extent, many participants used techniques to hide their birthmark, such as make-up, or sought treatment to reduce the stain: for example, laser therapy [130, 133]. In addition, the authors found that people with small PWS had exaggerated self-consciousness about their PWS compared to those with bigger marks [133]. In this regard, several participants reported having undergone many therapies during their childhood to treat their birthmark [132].

## Discussion

The main objectives of this systematic literature review were (1) to explore the impact of RGSD on daily life, emotional state, self-perception, and QoL; (2) to identify the main predictors of these health outcomes; and (3) to identify which characteristics in the psychosocial experience of the subjects are common to each of these various pathologies. We therefore focused on four RGSDs in this review: albinism, NF1, ichthyosis, and birthmarks. Of the 9987 recorded references, 48 were retained for analysis.

Despite deep differences in the biological and physiological mechanisms of the RGSDs we investigated, several common elements have emerged, related to their psychosocial impact. The common core of the main results observed in this review is represented in the following table (Table 6).

**Table 6:**
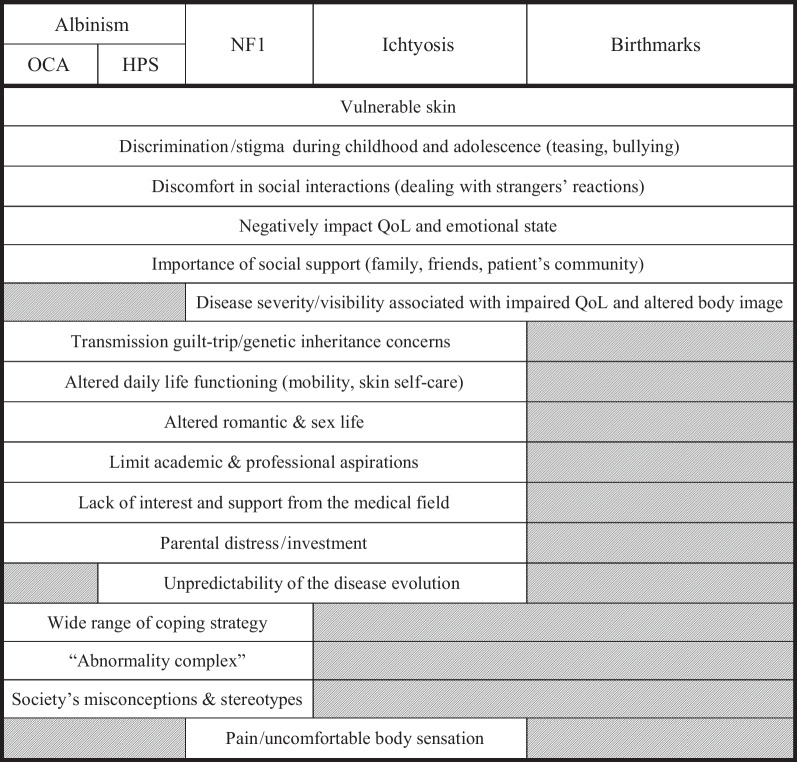
Summary table of results observed and common to at least two diseases

Firstly, the results of our investigation show that each RGSD has an impact on patients’ social life. Childhood and especially adolescence are pivotal periods in a person's development, where many changes (physical, cognitive, and social) occur and peers play a predominant role in the social development of the individual, largely influencing their own self-perception and identity construction [134, 135]. Unfortunately, according to the results, this is also the period when subjects are the most exposed to discrimination and stigmatizing experiences. Having a skin disease, a visible difference to others at this stage of life, would therefore favor the construction of a disabled or a stigmatized identity [136] that negatively impacts self-perception and social functioning. To illustrate this point, we can cite the study by Jensen et al. (2018) which showed that adolescence is the period of life when enacted and perceived stigma slides into more internalized stigma.

One of the relevant reviews conducted by Wanitphakdeedecha and colleagues (2021) aimed to explore what impact PWS had on self-stigma. As the authors remind us, self-stigma differs from social stigma in that it is an internalized mechanism in the stigmatized person who is anticipating social rejection. Self-stigma consists of three dimensions: perceived, anticipated, and enacted stigma.^4^ According to their findings, the negative impact of PWS on their psychosocial condition came mostly from patients’ enacted stigma or experiences with discriminatory acts toward their skin condition. On the other hand, perceived and anticipated stigma varied a lot among different age groups, knowing that anticipated stigma tends to increase with age. One of the most striking findings is that parents of PWS patients are more affected by self-stigma than their children. The psychological burden that this entails can however be regulated by good medical support and parent-health staff communication.

As a result, in all the conditions we have identified, participants expressed discomfort in social interactions, especially when they had to deal with strangers' reactions [37, 89, 95, 115, 117, 119, 121, 122, 130, 133].

However, although these diseases can cause social difficulties, these seemed to be compensated by the support patients receive from family, friends, or other patients involved in the same associations or organizations [31, 33, 37–39, 44, 89, 110, 111, 117, 119, 120, 133]. Indeed, social or family support was shown to be an important factor buffering the negative effects of stress in a wide range of clinical populations, for example, in the case of chronic skin conditions [137, 138] or in more critical health issues such as cancer [139–142]. However, some conditions within a specific context (in this case, albinism in Africa) can induce a very ambivalent family functioning mixing fear, protection, guilt, and violence [41].

Secondly, for each of the RGSDs investigated, patients’ QoL and emotional state were impaired. Very different determinants have been considered, but disease severity and visibility were the variables that researchers most often investigated. Many studies on NF1, ichthyosis, and birthmarks were able to show a link between the visibility or severity of the disorder, and a reduced QoL, depressive or anxious states, and an altered body image. These results echo other research conducted in subjects with chronic skin conditions such as atopic dermatitis [143–145], psoriasis [146–151] or vitiligo [152–156]. Other studies, conducted with "disfigured" patients, also resonate with our results [157]: we can mention craniofacial conditions, such as cleft lip or palate [158, 159], or even people with burn injuries [160, 161]. However, we can assume that there are differences in the impact that these different diseases have on body image. A line of thought is given to us by Ben-Tovim & Walker (1995; PWS and other chronic diseases), who suggest that women with visible diseases from birth or early childhood would be less prone to developing negative body attitudes than those acquiring visible illnesses during adolescence or adulthood. In order to cope with the visible nature of their condition, patients with NF1, ichthyosis, and birthmarks tried to camouflage their body parts that are most affected (clothing, make-up, tattoos) [119, 120, 122, 130, 133, 162]. These body-hiding techniques have shown their benefits on QoL in other visible conditions as vitiligo [163, 164], burn injuries [165, 166], facial skin disorders (acne, atopic dermatitis, psoriasis) [167, 168], infantile hemangiomas [169], and in post-surgical cancer treatment [170–172], which again demonstrates the impact of these conditions on body image [170, 172]. Furthermore, in the case of PWS, having had an early birthmark resorption treatment (e.g., flash-lamp pulsed dye laser) has a positive effect on the subject’s QoL [109]. Although many studies have shown the benefit of cosmetic camouflage in patients with skin disfigurement [173], another research has suggested that hiding any type of stigmatizing identity could have a negative impact on physical and psychological QoL [174]. Thus, the skin is a highly symbolically invested organ [175] and such genetic pathologies, like any visible disease, seem to induce stigmatization and social isolation, which are sources of anxiety, depressive disorders, emotional distress, impairment of QoL, and withdrawal [176].

In a surprising way, albinism is, in this study, the only genetic condition for which body image, and the visibility and severity of disease were not assessed. In PWA, low vision and QoL were the issues that Western researchers focused on most [177, 178]. In sharp contrast with the African continent, studies focused more on issues of discrimination linked to local beliefs and superstitions [19–22]. Many papers reported on the violence suffered by young PWA who were stalked, assaulted, or even killed for their physical attributes (hair, bones, etc.), particularly in Tanzania and Malawi. Furthermore, Africa has a majority dark-skinned population, making albinism all the more visible and therefore stigmatizing in the social environment. As we can see, the geographical and cultural context is a decisive factor in the understanding of this condition; health issues are drastically higher in Africa than in the rest of the world.

Considering this, it can also be noted that albinism, NF1, and ichthyosis are similar on several other issues, including the impact of the disease on daily life [31, 89, 92, 122, 128], on love life and sexuality [31, 37, 92, 110, 111, 115, 122], on academic and professional aspirations [31, 37, 111, 115, 122, 128], and finally on the lack of knowledge and interest from the medical community [39, 92, 112, 119]. Firstly, we can observe that impact on daily life greatly varies depending on the disease and the severity of the associated symptoms. In albinism, low vision seems to restrict patients' mobility as they are not allowed to drive a car [31], whereas in ichthyosis and NF1, unpleasant body sensations and pain disturb patients' sleep and walking [89, 92, 120, 128]. Thus, even though these rare diseases fit into the same taxonomic category, the complexity of their particularities invites us to distinguish them according to the specificities of their psychosocial impact. Mobility issues are also a widely identified difficulty in visual impairments such as glaucoma [179, 180], age-related macular degeneration (AMD) [181, 182], and diabetic retinopathy [183, 184]. Therefore, in terms of daily functioning, albinism is also comparable to ophthalmological conditions. However, there is one aspect on which all of these conditions converge: the importance of taking care of one's skin to avoid discomfort and complications. This long-term preventive treatment seems to play an important role in patients' daily lives.

These three genetic conditions also seemed to have a negative impact on patients' love and sex lives [31, 37, 92, 110, 111, 115, 122]. Studies on other visible conditions have observed similar results such as vitiligo [185], or in the case of severe burns [186]. The skin is an undeniable vector of intimacy and sexuality; when it is altered, body image is disturbed, and concerns about appearance and touch can make intimate relationships difficult.

Albinism and NF1 also share various other problems. Research showed a wide variety of adaptive strategies used by patients: use of repartee and humor in awkward social situations, scientific knowledge seeking, fighting spirit, spirituality, patient community involvement, physical hypervigilance, etc. [31, 32, 39, 111, 112, 120, 121]. These coping strategies are directly related to the fact that patients must constantly adapt themselves to societal norms in order to be seen as “normal”. Some even said they seemed to experience an "abnormality complex" when comparing themselves to others, both in terms of ability and learning [31, 33, 42, 112, 119]. For some people, there is a compelling need to conform, even if it means forcing themselves to pretend that they don’t have a disability (e.g., a child pretending to be able to read on the blackboard when they cannot). This type of behavior requires considerable effort from the person who gradually becomes exhausted and therefore weakens. Other studies revealed similar results in patients with intellectual disabilities [187, 188] and visual impairments [189]. Indeed, depending on the degree to which their impairment is mild or severe, people with physical or sensory impairments might attempt to overcome their difficulties in order to be considered as “normal” [136].

Finally, research has highlighted the distress experienced by some parents of children with NF1 or albinism. In this regard, parental stress and family functioning have been extensively studied in parents of children with developmental or intellectual disabilities [190] and sensory or physical impairments [191]. However, in the context of RGSD, parental distress has been supplemented by another concept. For all these conditions (with the exception of PWS), the prominence of guilt in the transmission of genetic disease is also notable. This notion has already been discussed by Chaumet in 2006, whose study explains that although the genetic disease may strengthen a sense of family belonging, it also makes the parent(s) feel guilty about giving the child a difficult and / or challenging life. Thus, choosing to have a child or not when affected by one of these genetic conditions becomes a real individual and family issue.

## Limitations

One of the main limitations of this review is the very attempt to synthesize common findings when the genetic conditions cover a wide range of different symptoms. Even if skin gathers these diseases within the same category, the skin is not affected in the same way in the case of albinism (hypopigmentation, photosensitivity) or NF1 (café-au-lait spots, neurofibromas). Moreover, some of these conditions are divided into subcategories according to the phenotypic or genotypic specificities of each. For example, albinism may be ocular, oculocutaneous, or syndromic, and the related psychosocial issues may vary from one form to another. One of the main differences identified in this review concerns OCA and HPS; syndromic forms of albinism which cause physiological disturbances (coagulopathy, an increased risk of neutropenia, pulmonary fibrosis, or Crohn's disease) that are not found in non-syndromic forms [192]. This aspect of the disease is an additional concern for HPS patients, who are sometimes worried about the unpredictable nature of the disease’s evolution [39].

Secondly, the methodological constraints inherent to the field of rare diseases make the generalization of these results almost impossible. The fact that these genetic diseases are very poorly represented in the general population makes it difficult to recruit voluntary participants. Sample sizes are often too small and suffer from representativeness bias. Even if some of these studies show significant results, the vast majority cannot generalize their observations. This results in great heterogeneity in study designs, sampling, and data collected, making it difficult to harmonize our findings. It should also be noted that the geographical context in which these studies were carried out has a major influence on the results, particularly in the case of albinism, and makes the synthesis of knowledge even more complex.

### Perspectives

One of the recurrent themes that we have not detailed above concerns the lack of interest and knowledge of doctors regarding RGSDs [31, 39, 92, 112, 119]. In this respect, it is important to note that rare diseases have never attracted as much interest around the world as they do today. In the last 20 years, medical findings in genetics have grown steadily, have improved diagnostic techniques, and deepened our understanding of how many diseases work. However, there are still large grey areas, and institutional and practitioner priorities remain focused on more common or more severe diseases (cancer, diabetes, cardiovascular diseases, dementia, etc.). As mentioned in our introduction, today 6000 to 8000 rare diseases have been identified in the world, each of them having a very particular functioning, more or less complex depending on the case. It is therefore almost impossible to be an expert on all of these diseases and to know how to take care of these patients. This issue can be illustrated by the fact that, in France, several patients with a rare disease experience significant diagnostic wandering, which can last up to six years after the first symptoms appear [2]. If physicians are in difficulty because they have scarce knowledge of these conditions, would they perhaps express a lack of interest in order to absolve themselves of responsibility for their medical follow-up?

At the medical level, genetic diseases raise a thorny ethical question regarding prenatal diagnosis. When parents know they are carriers of a potentially transmissible genetic anomaly, in particular contexts, they can resort to prenatal diagnosis and even selective termination of pregnancy if the fetus is affected. Clarke (2013) [193] addressed this question in families with Hypohidrotic Ectodermal Dysplasia and showed that opinions were divided among the participants; some were in favor of using these medical techniques, others against. This gap in opinion may then become a central issue for the parental couple who will have to make a crucial decision for their future. These ethical and legal questions have already been raised in the context of trisomy 21, in particular regarding the proximity that certain practices have with eugenics [194–196].

Secondly, in order to optimize healthcare, we could imagine developing a care plan that would begin as soon as the person is diagnosed. In fact, setting up a system with healthcare support time from when the diagnosis is first made would provide patients with important information about their condition, and would allow them to be oriented in the best healthcare pathway. These care systems have already shown their benefits when a serious illness, such as cancer, is diagnosed [197]. With this aim in mind, we can cite a French and a Korean team that have recently developed guidelines to help practitioners in the diagnosis and care of albinism [198] and NF1 [199]. In addition, practitioners could also offer patients the opportunity to participate in therapeutic education (TPE) sessions to learn how to manage their symptoms and develop coping strategies that would be useful in their daily lives. Some hospitals have already started to set up such a programme. For example, another French team has also developed a TPE protocol for children and adolescents with albinism and their parents [200].

This last element clearly highlights the need to offer patients biopsychosocial support based on a systemic approach. In view of the main results of this review, the whole family must be considered in patient follow-up, both at a genetic level (genetic inheritance pattern) and at a psychosocial level (psychoeducation, transmission guilt, coping with stigma). As Chaumet said in 2006, "genetic diseases are family affairs". Moreover, caring for patients with genetic diseases is a complex, time-consuming task that requires a holistic approach toward the patient and interdisciplinary support (links between geneticists, specialists in affected organs [such as the skin], and general practitioners). In order to better understand RGSDs, Bronfenbrenner's ecosystemic model (1979) [201] offers, in our opinion, the most appropriate approach for understanding and supporting such diseases. Bronfenbrenner (1979) maintains that the development of a person must be understood within a complex environmental system in which each system is conceived as a unit communicating with a larger and more organized system. Understanding the functioning of these different systems would therefore allow a better understanding of a patient case and thus promote their well-being. Speaking of a systemic approach, researchers have already proposed a global, cross-cutting framework for health stigma and discrimination. Based on theory, research, and practice, this framework demonstrates its application to a range of health conditions, including leprosy, epilepsy, mental health, cancer, HIV, and obesity/overweight [202]. It would therefore be very interesting to test it in future research on rare skin conditions, or to propose psychosocial interventions based on their findings. Concerning albinism, some French organizations specialized in sensory disabilities already seem to provide interesting interdisciplinary support to young PWA. For example, Assistance Services for Autonomy Acquisition and Schooling (in French: Service d'Aide à l'Acquisition de l'Autonomie et à la Scolarisation) and Early Medical and Social Action Centers (in French: Centres d'Action Médico-Sociale Précoce) work with many actors in order to help families concerned by albinism (or any other ophthalmologic genetic disease); health professionals are increasingly trained in systemic approaches and tend to approach care with transdisciplinarity [203, 204]. It would be judicious to see to what extent related medical, educational, and social establishments (Specialized Education and Home-Care Services; in French: Service d'Éducation Spéciale et de Soins à Domicile) could support families concerned by RGSD.

Finally, it is still essential to keep investigating the experiences of these populations, given the lack of evidence on these health issues in many countries. In order to compensate for sample size issues and to have more robust studies designs, it would be wise to set up longitudinal lifespan studies to see the possible developmental pathways in these diseases more precisely, and which factors would favour one pathway or another. In addition, action research or intervention-based studies could also be considered (prevention in schools, consultation/therapy for parents, etc.) and allow the implementation of direct and beneficial interventions for patients and their families.

## Conclusion

Despite the heterogeneity and methodological weaknesses of the studies we have investigated, this review has shed useful light on the psychosocial consequences of RGSDs on affected people and their families. In addition, the care pathway remains complex and tedious, and the professionals involved are few. Although new care systems are slowly being put in place in some institutes, access to adapted care remains limited and unequally distributed across countries. It would therefore be relevant to mobilize public policies in order to give more resources to associations and medico-socio educational centers that already support concerned families. As we have seen above, a systemic approach to the patient, considering all aspects of their life and environment (family, school, work, activities of daily living, etc.) would be the most optimal way to deal with these conditions.

## Supplementary Information

**Additional file 1:** Evaluation grid for qualitative exploratory studies.

## Data Availability

Not applicable.

## Abbreviations

DoK: Disorders of keratinisation
HPS: Hermansly-Pudlak syndrome
NF1: Neurofibromatosis type 1
PNf: Plexiform neurofibroma
PWA: People with albinism
PWS: Port-wine-stains
QoL: Quality of life
RGSD: Rare genetic skin disease
TPE: Therapeutic patient education

## Footnotes

1 Other diseases were also considered (primary cutaneous amyloidosis, tuberous sclerosis complex, epidermolysis bullosa, xeroderma pigmentosum) but were not selected for the following reasons: accompanied by too severe or too varied symptoms, no relevant studies, or a review of the literature already published (for more details, see “Method”).

2 New genes associated with albinism are still being discovered, both in its oculocutaneous form (OCA8) (Pennamen et al. 2021) and in its syndromic forms (HPS11) (Michaud et al. 2021).

3 Puerto Rico has a large population of people with Hermansky-Pudlak syndrome (HPS): where the global prevalence of HPS is 1–9/1,000,000, in Puerto Rico it drops to 1/1,800 (or almost 556 × larger).

4 Perceived stigma is an individual’s understanding of how others may think about and act toward an individual with different physical traits. Anticipated stigma refers to expectations of stigma in the future. Enacted stigma denotes discriminatory acts or behaviors experienced by the stigmatized people (Wanitphakdeedecha et al. 2021).
