# Supplementary material for: Psychosocial implications of rare genetic skin diseases affecting appearance on daily life experiences, emotional state, self-perception and quality of life in adults: a systematic review
Source: Orphanet J Rare Dis. 2023 Feb 23;18:39. doi: 10.1186/s13023-023-02629-1 (PMC9951542; doi:10.1186/s13023-023-02629-1)
Supplement: Supplementary file 1 — Additional file 1: Evaluation grid for qualitative exploratory studies. [file 13023_2023_2629_MOESM1_ESM.docx]

EVALUATION GRID FOR QUALITATIVE EXPLORATORY STUDIES

| THEORETICAL FRAME & RESEARCH QUESTION | |
| --- | --- |
| Is the research question clearly defined? (what problem?* what population?* what indicator(s)?*) | / 1,5 pt |
| Is the question justified by a coherent theoretical or empirical framework?  (where does it come from?* put into perspective with other work?*) | / 1 pt |
| METHODOLOGY | |
| Research protocol | |
| Is the context of the research sufficiently described (locations?* recruitment date range ?* recruitment method?*) | / 1,5 pt |
| Are the inclusion/exclusion criteria explicitly specified?** | / 1 pt |
| Is each step of the research well described (construction of the tools*, data collection process*, theoretical support to justify these methodological choices*) | / 2 pt |
| Are the tools used sufficiently described* and reliable* ? | / 1 pt |
| Is the question of results’ strength addressed?* How is it concretely considered in the protocol?* (e.g., thematic saturation) | / 1 pt |
| Data analysis | |
| Is the data analysis method sufficiently described* and justified (theory)* | / 1 pt |
| Is the analysis repeated by other independent researchers?** | / 1 pt |
| Do the researchers have the appropriate equipment to collect and process the data? (recorder? camera?...)* | / 0,5 pt |
| RESULTS | |
| Has the participation rate been addressed (number at recruitment, at eligibility,  included in the study, completed follow-up and included in the final analysis)?** | / 1 pt |
| Are the characteristics of the population (socio-demographic data) sufficiently described?** | / 1 pt |
| Do the researchers support their findings through the use of citations (or detailed descriptions)?** | / 1 pt |
| Can the results be considered strong (sufficient number of observations; saturation of themes...)?** | / 1 pt |
| Are results that contradict the original hypotheses (or contradict each other) addressed? Are the authors trying to find an explanation for them?* | / 1 pt |
| DISCUSSION | |
| Is the discussion/conclusion of the study consistent with the key findings?** | / 1 pt |
| Is the consistency of the results with other studies discussed?** | / 1 pt |
| Do the authors identify limitations of their study?** | / 1 pt |
| Do the authors identify perspectives for future research?* for clinical practice?* | / 1 pt |
| OTHER CONSIDERATIONS | |
| Is the funding (or lack thereof) of the study addressed? | / 0,5 pt |
| Are conflicts of interest addressed? | / 0,5 pt |
| Has the study been reviewed by an ethics committee | / 0,5 pt |

| TOTAL | /22 |
| --- | --- |
| PERCENTAGE | % |
| APPRECIATION | High / Moderate / Low |
